# Supplementary material for: Impact of doping on the carrier dynamics in graphene
Source: Sci Rep. 2015 Nov 18;5:16841. doi: 10.1038/srep16841 (PMC4649495; doi:10.1038/srep16841)
Supplement: Supplementary Information [file srep16841-s1.pdf]

Supplementary Material: Impact of doping on the carrier dynamics in graphene

Faris Kadi[1], Torben Winzer[1], Andreas Knorr[1], and Ermin Malic[2*]

[1]Institut für Theoretische Physik, Technische Universität Berlin, Hardenbergstr. 36, 10623 Berlin, Germany
[2]Chalmers University of Technology, Department of Applied Physics, SE-412 96 Gothenburg, Sweden
[*]ermin.malic@chalmers.se

ABSTRACT

Here, we introduce a semi-analytic approach to purely Coulomb-induced carrier multiplication to be able to study its fluence and doping dependence. Furthermore, we discuss the impact of the dynamical screening on the Coulomb interaction in doped graphene samples.

Semi-analytic approach to purely Coulomb-induced carrier multiplication

To be able to find optimal excitation conditions and the doping regime for (hot) carrier multiplication, we present a semi-analytical approach considering the purely Coulomb-induced carrier dynamics within statistical methods.[1]

Here, we focus on the carrier multiplication (CM), but the processes of *hot* carrier multiplication can be treated in an analogous way. Our approach is to evaluate the total carrier density n and the energy density $\mathscr{E}$ of the electronic system:

$$n = \frac{\sigma_s \sigma_v}{L^2} \sum_{\lambda=e,h;\mathbf{k}} \rho^{\lambda}_{\mathbf{k}} \;\text{ and }\; \mathscr{E} = \frac{\sigma_s \sigma_v}{L^2} \sum_{\lambda \mathbf{k}} \varepsilon^{\lambda}_{\mathbf{k}} \rho^{\lambda}_{\mathbf{k}}. \tag{1}$$

Assuming a Fermi distribution with $\varepsilon_F \neq 0$ for the carrier occupation $\rho^{\lambda}_{\mathbf{k}}$, we obtain the carrier and energy density as a function of the temperature T and the Fermi level ε_F:

$$n(T,\varepsilon_F) = \frac{\sigma_s \sigma_v}{2\pi} \sum_{\sigma=\pm} \int_0^{\infty} \mathrm{d}k \frac{k}{e^{(kv_F+\sigma\varepsilon_F)/(k_B T)}+1} = -\frac{2k_B^2}{\pi v_F^2} T^2 \sum_{\sigma=\pm} Li_2(-e^{\sigma \varepsilon_F / T k_B}), \tag{2}$$

$$\mathscr{E}(T,\varepsilon_F) = \frac{\sigma_s \sigma_v v_F}{2\pi} \sum_{\sigma=\pm} \int_0^{\infty} \mathrm{d}k \frac{k^2}{e^{(kv_F+\sigma\varepsilon_F)/(k_B T)}+1} = -\frac{4k_B^3}{\pi v_F^2} T^3 \sum_{\sigma=\pm} Li_3(-e^{\sigma \varepsilon_F / T k_B}), \tag{3}$$

where $Li_n(.)$ denotes the polylogarithm function.

After an optical excitation, the electronic system is described by the carrier density $n_{exc} = n_0 + n_{\text{opt}}$ and the energy density $\mathscr{E}_{exc} = \mathscr{E}_0 + \mathscr{E}_{\text{abs}}$. Here, $\mathscr{E}_{\text{abs}}$ denotes the absorbed pump fluence and $n_{opt} = 2\mathscr{E}_{\text{abs}}/\hbar\omega$ the optically excited carrier density with the photon energy $\hbar\omega$. Furthermore, the thermal densities $n_0 = n(T_0, \varepsilon_F)$ and $\mathscr{E}_0 = \mathscr{E}(T_0, \varepsilon_F)$ are determined by the initial temperature T_0 and Fermi level ε_F. To access the CM factor we need to know the final state of the carrier system after the Coulomb-induced thermalization. We exploit the fact that the Coulomb dynamics conserves the total energy density. Due to Auger processes, the total carrier density is not conserved, but instead the Fermi level. Using Eq. (3), we are able extract the final temperature T_f of the hot thermalized system by numerical variation with the condition that $\mathscr{E}(T_f, \varepsilon_F) = \mathscr{E}_{exc}$. Having the final temperature, we can also access the final carrier density $n_f = n(T_f, \varepsilon_F)$ and therewith also the CM factor, cf. Eq. (1) in the main manuscript. For undoped graphene, the purely Coulomb-induced T_f, n_f, and CM can be obtained analytically.[1] Note however that the approach does not include carrier-phonon scattering, which cannot be treated statically. Nevertheless, Fig. 1 shows an excellent agreement between the analytically [cf. Eqs. (2)-(3)] and numerically [cf. Eqs. (1)-(3) in the main part] obtained doping-dependent carrier multiplication at a fixed pump fluence of $\varepsilon_{\text{abs}} = 0.1\,\mu\text{Jcm}^{-2}$. The higher the Fermi level, the smaller is CM, and the larger is hCM. For highly doped graphene samples with $\varepsilon_F > 0.4\,\text{eV}$, CM becomes smaller than 1. In contrast, hCM reaches values of almost 5. The same qualitative dependence on doping is found by numerically evaluating the graphene Bloch equations (shown in the main part), which also include the carrier-phonon coupling. This demonstrates that the presented semi-analytical approach already sufficiently covers the most important aspects of doping-dependent carrier multiplication. For quantitative insights and time-dependent CM, the full microscopic approach is required.

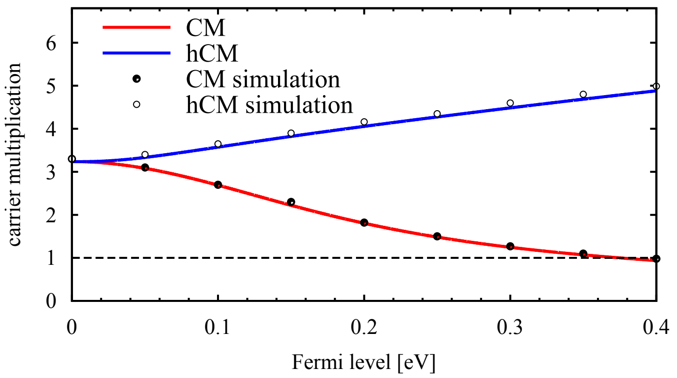

Figure 1. Analytically obtained CM and hCM as a function of doping at a fixed pump fluence (solid line) of $\varepsilon_{abs} = 0.1\,\mu\text{Jcm}^{-2}$ compared to the numerical solution (dots) obtained by evaluating the graphene Bloch equations presented in the main manuscript.

Fluence dependence of carrier multiplication

Now, based on the semi-analytical approach presented in the last section, we discuss the impact of the excitation strength on the appearance of the CM and hCM. Figures 2(a) and 3(a) show surface plots illustrating doping and fluence dependence of CM and hCM at 300 K and at an excitation energy of 1.5 eV, respectively. Our calculations demonstrate that both CM and hCM are strongly suppressed in the strong excitation regime. For pump fluences higher than approximately $5\,\mu\text{Jcm}^{-2}$ (hot) carrier multiplication does not occur any more. The larger the pump fluence, the more scattering partners are available giving rise to a faster carrier dynamics.[2] As a result, the thermalized distribution is reached within the first tens of fs and the asymmetry of

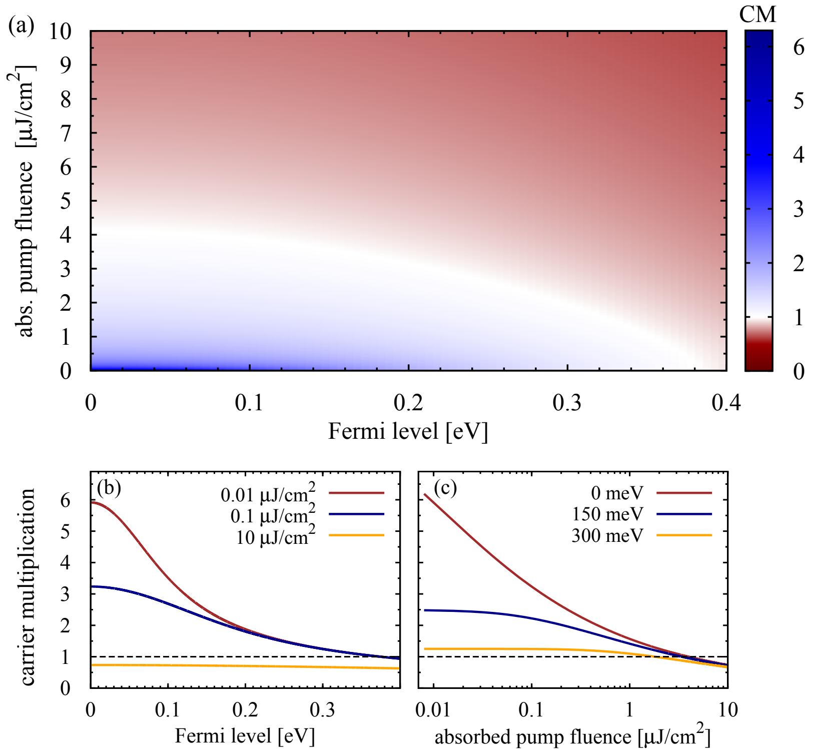

Figure 2. (a) Carrier multiplication (CM) as a function of Fermi level and pump fluence at room temperature and at an excitation energy of 1.5 eV. (b) Doping-dependent CM for three representative pump fluences and (c) fluence-dependent CM for three fixed Fermi levels.

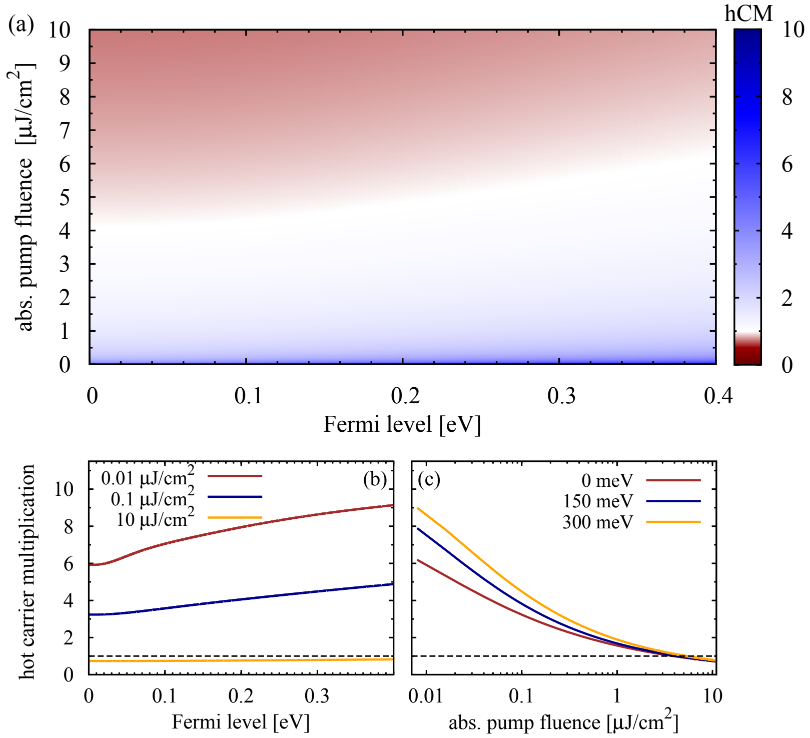

Figure 3. The same as in Fig. 2, however illustrating doping and fluence dependence of the hot carrier multiplication (hCM).

impact excitation and Auger recombination rates vanishes much faster. As a result, carrier multiplication clearly decreases at enhanced pump fluences, cf. Figs. 2(c) and 3(c) Furthermore, Figs. 2(b) and 3(b) also illustrate the decrease (increase) of CM (hCM) with the Fermi level, as already discussed in the main manuscript.

Dynamical screening

The efficiency of Auger processes is sensitive to the dynamical screening of the Coulomb interaction that is expressed by the Lindhard equation:[3]

$$\varepsilon(\mathbf{q},\omega) = 1 - 2V_{\mathbf{q}} \sum_{\lambda\lambda'\mathbf{k}} \frac{\rho^{\lambda'}_{\mathbf{k+q}} - \rho^{\lambda}_{\mathbf{k}}}{\varepsilon^{\lambda'}_{\mathbf{k+q}} - \varepsilon^{\lambda}_{\mathbf{k}} + \hbar\omega + i\delta^{\lambda}_{\mathbf{k,q}}} \Gamma^{\lambda\lambda'}_{\mathbf{k,q}}, \tag{4}$$

where ω corresponds to the energy transfer of the scattering processes and the factor $\Gamma^{\lambda\lambda'}_{\mathbf{k,q}} = |\int d^3r \Psi^{\lambda *}_{\mathbf{k}}(\mathbf{r}) e^{i\mathbf{q}\cdot\mathbf{r}} \Psi^{\lambda'}_{\mathbf{k-q}}(\mathbf{r})|^2$ includes the tight-binding wave functions $\Psi^{\lambda}_{\mathbf{k}}(\mathbf{r})$.

The appearing momentum-dependent dephasing $\delta^{\lambda}_{\mathbf{k,q}}$ can be derived from the dynamics of inhomogeneous charge fluctuations $\langle\tilde\rho_{\mathbf{q}}\rangle = -\frac{|e|}{L^3}\sum_{\mathbf{k}}\langle a^{\dagger}_{\mathbf{k-q}} a_{\mathbf{k}}\rangle = -\frac{|e|}{L^3}\sum_{\mathbf{k}}\sigma_{\mathbf{k-q,k}}$,. The corresponding equation of motion reads:[3]

$$\frac{d}{dt}\sigma_{\mathbf{k-q,k}} = \frac{i}{\hbar}(\varepsilon_{\mathbf{k-q}} - \varepsilon_{\mathbf{k}})\sigma_{\mathbf{k-q,k}} + \frac{iV_{\mathbf{q}}}{\hbar}(\rho_{\mathbf{k}} - \rho_{\mathbf{k-q}})\sum_{\mathbf{k}'}\sigma_{\mathbf{k'-q,k'}}. \tag{5}$$

Using the ansatz $\sigma_{\mathbf{k-q,k}}(t) = e^{-i(\omega+i\delta)t}\sigma_{\mathbf{k-q,k}}(0)$, a solution for $\sigma_{\mathbf{k-q,k}}$ can be obtained and leads to the dynamical screening expressed by the Lindhard formula with a fluence-dependent dephasing $\delta^{\lambda}_{\mathbf{k,q}}$, cf. Eq. (4) in the main text. To obtain the momentum-dependent $\delta^{\lambda}_{\mathbf{k,q}}$, we write the in-coherent equation of motion for the electron charge density in second-order Born-Markov approximation:[3]

$$\frac{d}{dt}\sigma_{\mathbf{k-q,k}}|_{nc} = -\frac{\pi}{\hbar}\sum_{ABCD}\tilde V^{AB}_{CD}\,\delta\left(\varepsilon_D + \varepsilon_C - \varepsilon_B - \varepsilon_A\right)\Big[\sum_{bcd}V^{\mathbf{k}b}_{cd}\left(\sigma_{\mathbf{k-q},D}\sigma_{bC}\sigma^{+}_{Ad}\sigma^{+}_{Bc} - \sigma_{Ad}\sigma_{Bc}\sigma^{+}_{\mathbf{k-q},D}\sigma^{+}_{bC}\right) - \sum_{abd}V^{a\ \ b}_{\mathbf{k-q}d}\left(\sigma_{aD}\sigma_{bC}\sigma^{+}_{Ad}\sigma^{+}_{B\mathbf{k}} - \sigma_{Ad}\sigma_{B\mathbf{k}}\sigma^{+}_{aD}\sigma^{+}_{bC}\right)\Big].$$

This equation contains the momentum-dependent dephasing term, which is given by $\frac{d}{dt}\sigma_{\mathbf{k-q},\mathbf{k}}|_{nc} \propto \delta_{\mathbf{k},\mathbf{q}}\sigma_{\mathbf{k-q},\mathbf{k}}$. Neglecting all polarization terms $\sigma_{ab} \to \rho_a$, the dephasing $\delta_{\mathbf{k},\mathbf{q}}$ yields:

$$\delta^{\lambda}_{\mathbf{k},\mathbf{q}} = S^{\text{in},\lambda}_{\mathbf{k},\mathbf{q}} + S^{\text{out},\lambda}_{\mathbf{k},\mathbf{q}} \tag{6}$$

with the in- and out-scattering rates

$$S^{\text{in},\lambda}_{\mathbf{k},\mathbf{q}} = \frac{\pi}{\hbar}\sum_{\mathbf{abc}}\left(V^{\mathbf{k}\ \lambda\ \mathbf{k}_c\lambda_c}_{\mathbf{k}_a\lambda_a\mathbf{k}_b\lambda_b}\tilde{V}^{\mathbf{k}_a\lambda_a\mathbf{k}_b\lambda_b}_{\mathbf{k}\ \ \lambda\ \mathbf{k}_c\lambda_c} + V^{\mathbf{k-q}\lambda\mathbf{k}_c\lambda_c}_{\mathbf{k}_a\lambda_a\mathbf{k}_b\lambda_b}\tilde{V}^{\mathbf{k}_a\lambda_a\mathbf{k}_b\lambda_b}_{\mathbf{k-q}\lambda\mathbf{k}_c\lambda_c}\right)[1-\rho_{\mathbf{a}}]\rho_{\mathbf{b}}\rho_{\mathbf{c}}\delta(\varepsilon^{\lambda}_{\mathbf{k}} + \varepsilon_{\mathbf{a}} + \varepsilon_{\mathbf{b}} + \varepsilon_{\mathbf{c}}),$$

$$S^{\text{out},\lambda}_{\mathbf{k},\mathbf{q}} = \frac{\pi}{\hbar}\sum_{\mathbf{abc}}\left(V^{\mathbf{k}\ \lambda\ \mathbf{k}_c\lambda_c}_{\mathbf{k}_a\lambda_a\mathbf{k}_b\lambda_b}\tilde{V}^{\mathbf{k}_a\lambda_a\mathbf{k}_b\lambda_b}_{\mathbf{k}\ \ \lambda\ \mathbf{k}_c\lambda_c} + V^{\mathbf{k-q}\lambda\mathbf{k}_c\lambda_c}_{\mathbf{k}_a\lambda_a\mathbf{k}_b\lambda_b}\tilde{V}^{\mathbf{k}_a\lambda_a\mathbf{k}_b\lambda_b}_{\mathbf{k-q}\lambda\mathbf{k}_c\lambda_c}\right)\rho_{\mathbf{a}}[1-\rho_{\mathbf{b}}][1-\rho_{\mathbf{c}}]\delta(\varepsilon^{\lambda}_{\mathbf{k}} + \varepsilon_{\mathbf{a}} + \varepsilon_{\mathbf{b}} + \varepsilon_{\mathbf{c}})$$

that also directly appear in the graphene Bloch equations, cf. Eq. (1) in the main part of the manuscript. Calculating these scattering rates, we have also a consistent access to the dephasing term influencing the dynamical screening of the Coulomb interaction. Note that a vanishing dephasing would suppress the parallel Auger scattering contributions in graphene due to a diverging $\varepsilon(\mathbf{q},\omega)$.[4] However, in realistic cases the many-particle processes lead to a significant dephasing opening up the Auger channels for the carrier relaxation.[5]

References

1. T. Winzer and E. Malić. Impact of auger processes on carrier dynamics in graphene. *Physical Review B* **85**, 241404 (2012).
2. T. Winzer, A. Knorr and E. Malic. Carrier multiplication in graphene. *Nano Letters* **10**, 4839–4843 (2010).
3. H. Haug and S. W. Koch. *Quantum Theory of the Optical and Electronic Properties of Semiconductors* (World Scientific, 2004).
4. D. Brida, A. Tomadin, C. Manzoni, Y. J. Kim, A. Lombardo, S. Milana, R. R. Nair, K. S. Novoselov, A. C. Ferrari, G. Cerullo and M. Polini. Ultrafast collinear scattering and carrier multiplication in graphene. *Nature Communications* **4**, 1987 (2013).
5. T. Winzer and E. Malic. The impact of pump fluence on carrier relaxation dynamics in optically excited graphene. *Journal of Physics: Condensed Matter* **25**, 054201 (2013).
